# Genomic prediction of crown rust resistance in *Lolium perenne*

**DOI:** 10.1186/s12863-018-0613-z

**Published:** 2018-05-29

**Authors:** Sai Krishna Arojju, Patrick Conaghan, Susanne Barth, Dan Milbourne, Michael D. Casler, Trevor R. Hodkinson, Thibauld Michel, Stephen L. Byrne

**Affiliations:** 1 Teagasc, Crop Science Department, Oak Park, Carlow, R93 XE12 Ireland; 2 Teagasc, Grassland Science Research Department, Animal and Grassland Research and Innovation Centre, Oak Park, Carlow, R93 XE12 Ireland; 3 Department of Agronomy, University of Wisconsin-Madison, Madison, WI53706 USA; 4 Agricultural Research Service, United State Department of Agriculture, Madison, WI53706 USA; 5 Department of Botany, School of Natural Sciences, Trinity College Dublin, Dublin 2, Ireland

**Keywords:** Genomic selection, Crown rust, Perennial ryegrass, Genetic relationship, GWAS

## Abstract

**Background:**

Genomic selection (GS) can accelerate genetic gains in breeding programmes by reducing the time it takes to complete a cycle of selection. *Puccinia coronata* f. sp *lolli* (crown rust) is one of the most widespread diseases of perennial ryegrass and can lead to reductions in yield, persistency and nutritional value. Here, we used a large perennial ryegrass population to assess the accuracy of using genome wide markers to predict crown rust resistance and to investigate the factors affecting predictive ability.

**Results:**

Using these data, predictive ability for crown rust resistance in the complete population reached a maximum of 0.52. Much of the predictive ability resulted from the ability of markers to capture genetic relationships among families within the training set, and reducing the marker density had little impact on predictive ability. Using permutation based variable importance measure and genome wide association studies (GWAS) to identify and rank markers enabled the identification of a small subset of SNPs that could achieve predictive abilities close to those achieved using the complete marker set.

**Conclusion:**

Using a GWAS to identify and rank markers enabled a small panel of markers to be identified that could achieve higher predictive ability than the same number of randomly selected markers, and predictive abilities close to those achieved with the entire marker set. This was particularly evident in a sub-population characterised by having on-average higher genome-wide linkage disequilibirum (LD). Higher predictive abilities with selected markers over random markers suggests they are in LD with QTL. Accuracy due to genetic relationships will decay rapidly over generations whereas accuracy due to LD will persist, which is advantageous for practical breeding applications.

**Electronic supplementary material:**

The online version of this article (10.1186/s12863-018-0613-z) contains supplementary material, which is available to authorized users.

## Background

Perennial ryegrass (*Lolium perenne* L.) is the predominant forage species grown in temperate regions of the world [1]. *Puccinia coronata* f. sp. *lolli* (crown rust) is one of the most widespread diseases of perennial ryegrass and can lead to a reduction in forage nutritive value, yield and persistency [2–4]. Poor quality, rust infected swards can impact animal performance and well-being [5–7]. Developing resistant cultivars is the most viable option for disease control and it has been shown that resistance to crown rust is conferred by both quantitative and qualitative inheritance [8–11]. As an obligate out-crossing species, perennial ryegrass germplasm has high variation for disease resistance that can be utilized to develop resistant cultivars [11–13]. Phenotypic recurrent selection is typically used to develop cultivars with improved resistance and selection is often carried out on spaced plants [9, 11, 12, 14]. There is a high correlation between spaced plants and swards for disease resistance and indirect selection for disease resistance on spaced plants can improve resistance in sward conditions [15]. However, with the advancements in molecular marker development over the last decade, efforts to use marker assisted breeding strategies have been pursued. One such strategy involves identifying quantitative trait loci (QTL) in bi-parental mapping populations and using markers to efficiently backcross the QTL into elite breeding material [16]. Although QTLs explaining significant phenotypic variation for crown rust resistance were mapped onto linkage group (LG) 1-5 and 7 [17–23], it is unclear if any of these QTLs were successfully introduced into breeding material. Genome wide association studies (GWAS) are another approach to identify markers linked to QTL. In this case breeding populations can directly be used to identify marker-trait associations, although identified markers tended to explain a small proportion of the total additive genetic variance, resulting in smaller genetic gains [24–26].

Genomic selection (GS) was first proposed by Meuwissen et al. [27], as a method to capture complete additive genetic variance using genome wide markers. GS is a form of marker assisted breeding, which accounts for all marker effects across the entire genome to calculate genomic estimated breeding values (GEBVs), which are used to select individual plants for advancement [26]. Use of genome-wide markers will include small effect loci and is ideal for complex traits with low to moderate heritability. In GS, a training population is genotyped with genome wide markers and phenotyped for the trait under selection and models to predict breeding values from marker data are developed. Implementing GS for complex traits like yield and quality is a primary objective of many perennial ryegrass breeding programmes. In contrast to yield and quality traits, the cost (labour and time) of phenotyping for disease resistance is much lower. However, it is important that any GS approaches targeting yield and quality improvements also ensure adequate disease resistance is maintained, particularly where multiple rounds of marker based selections are performed between field evaluations. Opportunities for GS in perennial ryegrass were first reviewed by Hayes et al. [28], and the earliest empirical study was done by Fè et al. [29] for heading date, which confirmed the superiority of GS over marker assisted selection. Later Fè et al. [30], Grinberg et al. [31] and Byrne et al. [32] reported high predictive ability for important agronomical traits in perennial ryegrass. In particular, predictive ability for crown rust reached up to 0.58 [30] when genotypes and phenotypes were evaluated on F_2_ families. In this study, we evaluated predictive ability for crown rust resistance on individual plants in a large perennial ryegrass population, and assessed factors contributing to predictive ability, such as training population size and marker density. We also performed a GWAS to identify a small to moderately sized panel of markers with good predictive ability for crown rust resistance.

## Methods

### Plant material, phenotyping and genotyping

The training population consists of 30 diploid perennial ryegrass families that have been described previously [32, 33]. Each family consists of 60 genotypes making up a population of 1800 individuals. The complete population consists of ten cultivars, eight full-sib families, eight half-sib families and four ecotypes. Plants were established in a glasshouse and later transplanted to the field in 2013 at Oak Park, Carlow, Ireland (52° 51^′^34.2^″^*N*, 6° 55^′^03.0^″^*W*). Plants were grown in two replicates in a partially balanced incomplete block design. Each block consists of 60 test genotypes and 5 check genotypes and was surrounded by a 1.5 m sward consisting of a four way mix of crown rust susceptible perennial ryegrass cultivars. Crown rust was recorded in the years 2014 and 2015 as mean percentage disease score on each plant. Briefly, percentage disease score was obtained by combining scores of percentage of leaves with infection and average percentage of infection on diseased leaves. Scoring was done at multiple time points in September to November without any harvest cuts between scorings (Table 1). We are trying to develop genomic models to identify plants with good resistance to crown rust across the season, and we decided to use all time points for constructing a quantitative summary for crown rust resistance. To do this we calculated AUDPC for each spaced plant in both years. Using multiple time point data, we generated area under disease progress curve (AUDPC) as follows:

1 $$ A_{k} = \sum_{i=1}^{N_{i}-1} \frac{(y_{i}+y_{i+1})}{2} (t_{i+1} - t_{i})   $$

**Table 1:** Mean percentage disease score for crown rust resistance at different time points (TP) in Year1 (2014) and Year2 (2015)

Time point/dates	Mean	SD	Min	Max
Year 1				
TP1 (13/10/14)	3.1	6.1	0	40
TP2 (20/10/14)	5.2	7.6	0	45
TP3 (29/10/14)	9.6	10.8	0	60
TP4 (10/11/14)	9.8	8.7	0	45
Year 2				
TP1 (21/09/15)	2.0	4.4	0	32
TP2 (05/10/15)	11.2	10.0	0	60
TP3 (19/10/15)	19.9	9.0	0	63

where *y*_*i*_ is the extent of infection (percentage disease score) at *i*^*th*^ observation and *t*_*i*_ is the time point at *i*^*th*^ observation. *N*_*i*_ is the number of individuals in the data set.

Variance components for crown rust were estimated using the R package lme4 (linear mixed-effects models using ’eigen’ and S4) [34]. Broad sense heritability was estimated as follows:

2 $$ H^{2} = \frac{\sigma_{g}^{2}}{\left(\sigma_{g}^{2}\right) + \left(\sigma_{g*yr}^{2}\right)/2 + \left(\sigma_{res}^{2}\right)/4}   $$

where $\sigma _{g}^{2}$ is the total genetic variance among individuals, $\sigma _{g*yr}^{2}$ is the variance associated with genotype by year interaction and $\sigma _{res}^{2}$ is residual variance. With genotype as random effect and year and checks as fixed effects, conditional modes (BLUPs) were calculated in lme4 and used as input for genomic prediction.

Genotyping was done using genotyping by sequencing (GBS) approach described by Elshire et al. [35] and data analysed as described in Byrne et al. [32]. Briefly, genomic DNA was extracted from leaf samples and GBS libraries were prepared using the restriction enzyme ApeKI, libraries were amplified and sequenced on an Illumina Hiseq2000. Panels of SNPs were identified in the complete population, as well as in all sub populations separately (half-sibs, full-sibs, ecotypes, cultivars). Individuals with very low sequencing coverage and/or largely missing phenotypic data were eliminated from the analysis giving a final population for analysis of 1582 individuals. Missing marker data was imputed using mean imputation.

### Genomic prediction models

We used four statistical algorithms for genomic prediction, ridge regression best linear unbiased prediction (rrBLUP) [27], Bayes B [36] and Bayesian Lasso [37], and random forest [38]. rrBLUP is a mixed model approach, which was initially proposed for GS. We used an R package called rrBLUP [39] for fitting the mixed model as follows

3 $$ y = \mu + Xg + \epsilon  $$

where *μ* is the overall mean, X is the marker matrix, g is the matrix of marker effects, *ε* is a vector of residual effects and y is a vector of conditional modes for crown rust. We also evaluated two Bayesian approaches, Bayes B [36] and Bayesian Lasso [37], which were both implemented using the R package BGLR [40] with the following parameters: number of iterations = 5000, burn-in = 500 and thinning = 5. Random forest is a machine-learning tool, in which series of regression trees were grown independently to the largest extent possible using subsets of bootstrap samples. At each split of the tree, a random subset of variables is selected to identify the best split. We implemented random forest using the R package randomForest [41], setting the number of variables at each split to 1/3 of the total variables, and using a terminal node size of five and minimum of 500 trees per forest. We also used random forest to rank variables using the variable importance measure, a permutation based measure in which variables are ranked based on the mean decrease in accuracy.

### Cross validation scheme

We evaluated genomic prediction models using Monte-Carlo cross-validation by randomly assigning plants into training (70%) and test (30%) sets and the procedure was repeated 100 times and the resulting accuracies were then averaged. This approach to cross-validation has previously been used to evaluate genomic prediction models [42, 43]. Predictive ability and bias were assessed in the complete population and in each sub-population. Predictive ability (*r*_*p*_) was determined as the Pearson’s correlation coefficient between observed phenotypic value and predicted phenotype. Bias was evaluated by regressing observed phenotypic value on predictions. We reduced training population size and marker density in order to identify the impact of training population size and marker number on predictive ability. To compare predictive ability for traits with contrasting genetic architecture we compared heading date, a highly heritable trait, with crown rust. Predictive ability for heading date has already been shown to be high (0.81) in this population [32]. We re-analyzed data for heading date according to methods described above and made a comparison with crown rust. To evaluate the impact of leaving related material out of the training set we also performed cross validation by leaving one family out. In this approach one complete family (up to 60 individuals) is left out of the training set and only used for testing. This was repeated so that each family in turn is used as a test set.

### Genome wide association

A mixed linear model (MLM) was also used for association mapping, implemented in the R package rrBLUP [39]. Population structure and family relatedness was accounted for with a kinship matrix calculated by rrBLUP from the input genotypic data. We accounted for multiple testing using a Bonferroni correction and markers passing an *α* level 0.05 threshold were considered statistically significant.

## Results and discussion

### Phenotypic analysis for crown rust

The mean percentage disease score for crown rust infection in the population increased over time in both evaluation years as infection levels accumulated (Table 1). In both years, evaluations were carried out in the period from September to November during a time when disease pressure tends to be at its greatest [15, 44]. The highest mean percentage disease score was seen in late October 2015 and was more than double the highest mean percentage disease score from 2014 (Table 1). In addition to plant health and level of host resistance, crown rust infection is influenced by various environmental factors, such as temperature, relative humidity, and light [45–47]. The latency period is reduced and spore production increased as temperature increases [45], and it has been shown that when temperatures exceed 25°C, the susceptibility of previously resistant cultivars can be increased [46]. It has already been shown that there is variability within pathogen populations, and different races can be found within and between locations. It is also possible that the composition of a pathogen population can change over short periods of time and plants that are resistant at one point in time will become susceptible as the pathogen population shifts or evolves.

**Fig. 1:**
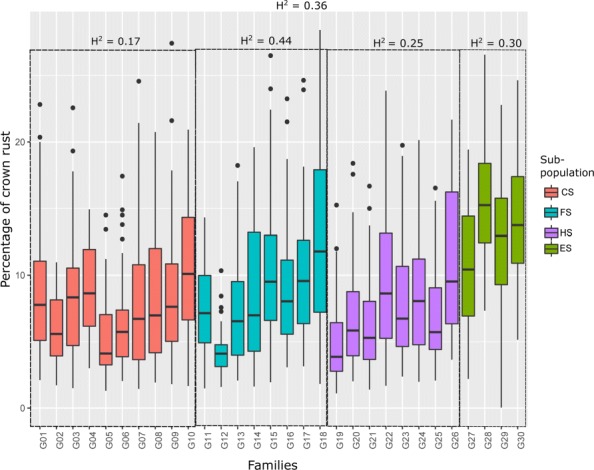
Phenotypic variation for crown rust resistance in the complete population, grouped according to sub-population types: cultivars (CS), ecotypes (ES), full-sibs (FS) and half-sibs (HS). Broad sense heritability (*H*^2^) in complete population and sub-populations is highlighted over the figure

AUDPC values ranged from 0 to 1371 and the Pearson correlation co-efficient between replicates within years was moderate (0.69 in 2014 and 0.59 in 2015). However, the Pearson correlation co-efficient between years was low (0.28), and there was a significant genotype by year interaction (*F*_(1761)_= 3.025, *MSE* = 60676, *p* = 0.0001). The broad sense heritability for crown rust infection was moderate (0.36), which is in line with previous estimates of heritability calculated in other populations [11, 48]. Overall there is a good phenotypic variation for crown rust infection among and within the 30 families/cultivars/ecotypes making up the entire population (Fig. 1). Plants were placed into one of four categories (sub-populations) based on mating type or origin, these were (i) full-sib families, (ii) half-sib families, (iii) cultivars, and (iv) ecotypes. In general the ecotypes were more susceptible to crown rust infection than cultivars or breeding material (Fig. 1), which presents a challenge for the incorporation of ecotypes into breeding programmes. The broad-sense heritability calculated in each sub-population varied between 0.17 in the cultivars to 0.44 in the full-sib families.

Crown rust infection is typically evaluated in breeding programmes by growing spaced plants or potted plants from a population and visually scoring the level of crown rust infection. A mean score is assigned to each family and used to aid selection of the top performing families from which to construct the synthetics cultivars. During construction of synthetics a spaced plant nursery may be established to evaluate heading date and crown rust resistance before selecting individual genotypes from which to construct synthetics (within family selection). In practice, this has a time cost of 2 to 3 years (establishment, evaluation, selection and recombining), and using molecular markers offers an opportunity to reduce this to one year in those selection cycles where GEBVs are predicted. This depends on our ability to accurately predict traits such as crown rust from genomic data.

### Predicting crown rust resistance with genomic data

We evaluated four algorithms for prediction of crown rust infection from genomic data, rrBLUP, Bayes B, Bayesian Lasso, and random forest. The mean predictive ability after cross-validation within the complete population was 0.52 using rrBLUP, 0.52 using Bayesian Lasso, 0.51 using Bayes B, and 0.49 using random forest (Additional file 1: Figure S1). rrBLUP was computationally faster, and therefore results from all further analysis are only reported for rrBLUP. The predictive ability of 0.52 is in line with previous estimates reported in perennial ryegrass where predictions were based on mean genotypes and phenotypes of F_2_ families [30]. Predictive ability did not differ depending on whether the equations were developed using phenotypes from the last time point scored or the AUDPC values incorporating all time points. This indicates that a single scoring each year would have sufficed. However, the importance of evaluating crown rust in more than one year was emphasised by the low correlation between scores in 2014 and 2015.

**Fig. 2:**
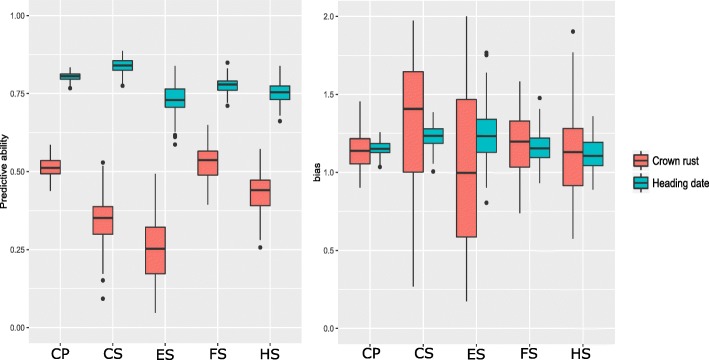
Predictive ability in different population types. Complete population (CP), cultivars (CS), ecotypes (ES), full-sibs (FS) and half-sibs (HS) are listed on x-axis, predictive ability (left) and bias (right) on y-axis. Crown rust is in red and heading date in blue

When we calculate the predictive ability within each of the sub-populations (cultivars, half-sib families, full-sib families, and ecotypes), the highest predictive ability for crown rust was obtained using plants from full-sib families (0.54) and the lowest predictive ability for crown rust was obtained with the plants from the ecotypes (0.24) (Fig. 2). Generally, traits with higher heritability achieve higher predictive abilities [49, 50], and we see that here where crown rust measurements taken in the full-sib families had the highest broad-sense heritability and the highest predictive ability. In general, there was a good correlation between predictive ability and both phenotypic variance and heritability. This relationship between phenotypic variance and predictive ability has been observed previously [49, 51].

We also evaluated the predictive ability using a leave-one-family-out cross validation scheme. The complete population is comprised of 30 families/cultivars/ecotypes, each with up to 60 individual genotypes. The predictive ability was assessed in the complete population by selectively leaving one family out of the training set and using it for testing. In addition to crown rust we also evaluated predictive ability for heading date phenotypes previously reported [32]. The predictive ability for both crown rust (*r*_*p*_=0.02, min =−0.36, max = 0.36) and heading date (*r*_*p*_=0.29, min =−0.14, max = 0.65) varied greatly depending on which family was left out, and having related material in the training set (shared parentage) greatly improved predictive ability.

### Effect of training population size and marker density on predictive ability

**Fig. 3:**
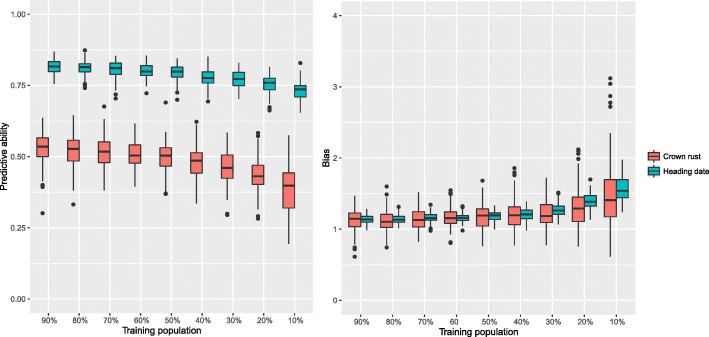
Effect of training population size on predictive ability. Training population is varied from 90% (1423 individuals) to 10% (158 individuals) on x-axis and predictive ability (left), bias (right) on y-axis. Crown rust is in red and heading date in blue

As we reduced the number of individuals in the training population we saw a decrease in predictive ability and an increasingly upward bias in the variance of predictions for both crown rust resistance and heading date (Fig. 3). The drop in predictive ability was more pronounced as we reduced the training population size for crown rust resistance than it was for heading date. The predictive ability for crown rust resistance when using 90% of the population as a training set was 0.52 and the predictive ability was 0.38 when using just 10% of the population. Irrespective of the trait, as the training population size increased there was an increase in predictive ability which is consistent with similar correlations between training population size and predictive ability reported previously for perennial ryegrass [29, 30] and other crops [51–54]. Useful linkage disequilibrium (LD) only extends over short distances in perennial ryegrass and it has been suggested that this is the result of a very large past effective population size [28]. This impacts both the size of the reference population and marker density required to achieve high accuracies when predicting traits from genomic data. The fact that we are able to achieve high predictive abilities with relatively small training populations is likely a result of strong genetic structure and differentiation in our diverse population and the use of the marker data to capture genetic relationships [55].

The limited LD also affects the number of markers required to obtain high predictive accuracies, and given the extent of LD in the broader perennial ryegrass population, marker numbers in excess of one million have been suggested for achieving high accuracies [28]. When we reduced marker number in the complete population and the various sub-populations we observed very little impact on the predictive ability for either trait (Table 2). Reducing the marker set to 5% of the total available had virtually no impact on predictive ability in all cases. This would support our observation that much of the predictive ability can be derived from makers capturing familial relationships. When marker number dropped below 5% (10878) predictive ability for both traits in the complete population began to drop. However, even with 0.05% (109) of markers the mean predictive ability was 0.30 for crown rust resistance and 0.52 for heading date. Knowing the contribution of genetic relationships to predictive ability is important because it will change over generations. In contrast, predictive ability due to LD has greater persistence over generations and is therefore preferential [55]. Schemes for implementing genomic selection in perennial ryegrass that pursue a reduction in effective population size from the outset have been proposed. Such schemes would lead to an increase in the extent of LD and ensure that predictive ability due to LD can be captured using a reasonable number of markers and a reference population size that is feasible in breeding programmes.

**Table 2:** Predictive ability (*r*_*p*_) and bias for crown rust (CR) and heading date (HD) by selecting random markers of 100 to 0.05%, in complete population (CP), cultivars (CS), full-sibs (FS) and half-sibs (HS)

Pop	100%		60%		20%		5%		1%		0.5%		0.1%		0.05%	
	*r*_*p*_	*bias*	*r*_*p*_	*bias*	*r*_*p*_	*bias*	*r*_*p*_	*bias*	*r*_*p*_	*bias*	*r*_*p*_	*bias*	*r*_*p*_	*bias*	*r*_*p*_	*bias*
CR																
CP	0.52	1.22	0.52	1.22	0.52	1.21	0.51	1.18	0.46	1.10	0.43	1.07	0.36	1.04	0.30	1.48
CS	0.29	1.28	0.28	1.26	0.28	1.24	0.27	1.18	0.22	0.97	0.17	0.80	0.14	0.95	0.10	1.13
FS	0.54	1.13	0.54	1.13	0.54	1.13	0.54	1.14	0.52	1.07	0.50	1.03	0.45	1.00	0.40	0.99
HS	0.49	1.24	0.49	1.24	0.49	1.24	0.49	1.24	0.48	1.23	0.46	1.21	0.42	1.23	0.36	1.22
HD																
CP	0.81	1.16	0.81	1.16	0.81	1.16	0.80	1.14	0.75	1.07	0.72	1.05	0.62	1.01	0.52	1.00
CS	0.84	1.25	0.81	1.19	0.81	1.20	0.81	1.18	0.78	1.11	0.77	1.12	0.66	1.03	0.56	1.02
FS	0.76	1.00	0.75	1.16	0.75	1.16	0.75	1.16	0.74	1.14	0.68	1.27	0.64	1.26	0.62	0.87
HS	0.74	1.18	0.74	1.09	0.74	1.10	0.74	1.09	0.73	1.08	0.72	1.15	0.67	1.10	0.62	1.09

### Identifying SNPs associated with crown rust resistance

**Fig. 4:**
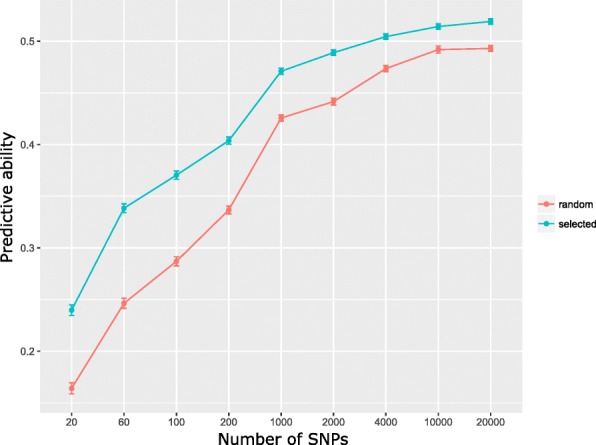
Predictive ability of selected markers versus random markers in the complete population. Markers were selected based on the ranking from genome wide association studies and compared with random markers of similar size

**Fig. 5:**
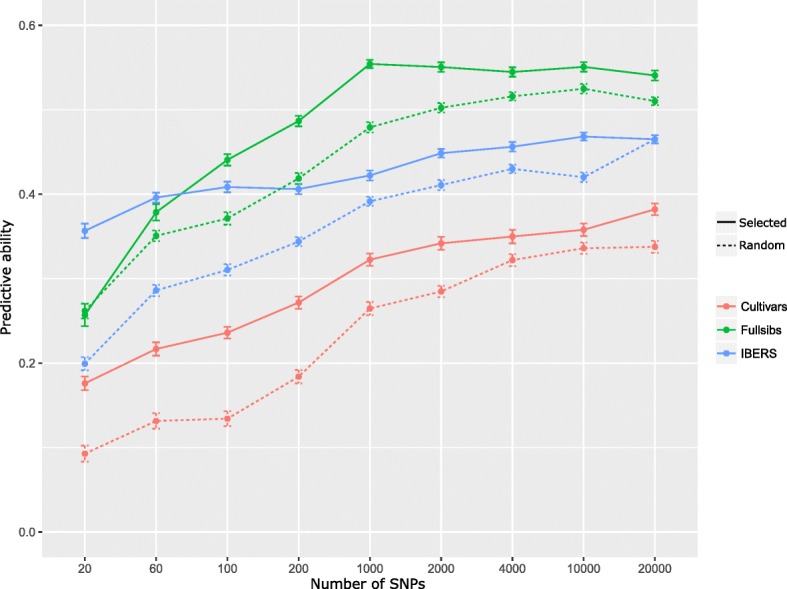
Comparing predictive ability of selected versus random markers. Markers were selected based on the ranking from genome wide association studies in cultivars, full-sibs and IBERS material and compared with random markers of similar size

The cost of genotyping impacts the number of selection candidates that can be evaluated and therefore impacts the selection intensity. Different approaches to low density SNP genotyping for genomic selection have been proposed. These include variable selection methods to identify a small subset of markers in strong LD with the trait [56] or using a small random subset of markers to impute from low-to-high density [57]. Until a chromosome scale assembly of the perennial ryegrass genome becomes available the latter remains a challenge. We used both permutation based variable importance measures and GWAS analysis to identify a subset of markers capable of predicting crown rust resistance. Using permutation based variable importance measures we were able to rank markers by mean decrease in accuracy and select the top ranked markers for use in genomic prediction. In the case of GWAS we ranked SNPs based on significance and again selected the top ranked markers for use in genomic prediction. All variable importance measures and GWAS were identified and ranked in the training set and used to predict phenotypes in the test set via cross-validation. When we used the top 100 ranked markers from the permutation based variable importance measures, the mean predictive ability of 100 iterations was 0.42 (ranging from 0.36 to 0.48). When we used the top 100 ranked markers from the GWAS analysis, the mean predictive ability of 100 iterations was 0.36 (ranging from 0.25 to 0.44). In both cases the mean predictive ability with selected markers is higher than the predictive ability with random markers, which was 0.28 (ranging from 0.18 to 0.39). The lower predictive ability using GWAS marker selection is not surprising considering that we corrected for population structure using a kinship matrix, and we are more reliant on identifying markers in LD with the trait. As discussed above, the predictive ability of these markers is expected to be more persistent over subsequent generations. Using GWAS selected markers it is clear to see that they are superior to randomly selected markers up to the point, beyond which adding more markers does not improve predictive ability in either case (Fig. 4). The ability of a GWAS within each sub-population to identify and select a small set of SNPs with excellent predictive ability varied, and in some cases was little better than random SNP selection (Fig. 5). The GWAS on plants originating from IBERs bred cultivars identified a small set of twenty SNPs with 77% of the predictive ability achieved with 20,000 SNPs. The power of a GWAS to identify markers with high predictive ability was much greater within the population made up of IBERs plants than within cultivars, and full-sib families where twenty SNPs could only achieve 46 and 48% of the predictive ability with 20,000 SNPs, respectively. On average LD is higher within the sub-population with IBERs plants, which may explain the greater ability to identify markers associated with crown-rust resistance.

In order to characterise the markers associated with crown rust resistance we repeated the GWAS analysis without division of genotypes into training and testing sets. We carried out GWAS using the complete population and found 29 markers significantly associated with crown rust resistance after correction for multiple testing (Additional file 2: Table S1). Using the perennial ryegrass genome [58] as a reference, we located all markers within 22 genomic scaffolds that contained 50 predicted genes. Using the Genome Zippper [58, 59], we anchored ten scaffolds onto LG2, 3, 4, 5 and 7 (Additional file 3: Table S2). Similarly, we did GWAS on IBERS material and found 24 markers associated with crown rust resistance (Additional file 2: Table S1). All markers were located within 16 genomic scaffolds containing 44 predicted genes. Out of 16 scaffolds we were able to place seven scaffolds onto LG3, 5 and 7 (Additional file 3: Table S2). We found five common scaffolds between the complete population and the IBERS and only two of these scaffolds were mapped, onto LG3. On LG3 five markers were anchored within 60.4-61.21 cM. Genes present on these scaffolds were coding for domains including Mon1, Aquaporin, DUF1635, Nucleoredoxin, Beta-glucan export ATP-binding/permease protein, BRASSINOSTEROID INSENSITIVE 1-associated receptor kinase 1, Alpha N-terminal protein methyltransferase 1. Gene function of these domains plays a key role in ATP-binding, membrane proteins, enzyme catalysis and pathogen-associated molecular pattern (PAMP)-triggered immunity (PTI) (Additional file 4: Table S3) [60].

Using small subsets of trait associated markers may be an effective strategy for within-family prediction of traits such as heading date, crown rust resistance and some quality traits. Predicting heading date from markers would enable plants to be matched in heading date to ensure sufficient cross-pollination when constructing synthetic cultivars [32]. Combining these with markers to predict crown rust resistance would also avoid the inclusion of plants with high levels of susceptibility, and furthermore prediction models can be based on multi-year evaluations. It is clear from the phenotypic data presented here that there is substantial within family variation for crown rust resistance. Opportunities already exist to genotype small to moderate sized marker panels in 1000s of samples at low cost [61]. Using these approaches small fragments (200-300 bp) are amplified and sequenced at hundreds of loci. These amplicons can be used as short haplotypes in marker aided selection strategies. An assay can be developed to target loci in linkage with QTL for heading date [32], crown rust resistance, and other traits such as quality parameters. The assay can also include a suite of loci with a good distribution throughout the genome and be deployed for among-and-within-full-sib-family selection (Additional file 5: Figure S2). Once high yielding families are identified in field trials, within family selection for crown rust resistance and forage quality can be performed at a high selection intensity with the molecular marker assay. Furthermore, plants can be selected to be synchronous in flowering time.

## Conclusions

Our findings show that predicting crown rust resistance in perennial ryegrass can be achieved with high accuracy using AUDPC scores on spaced plants. However, there was no difference in predictive ability when equations were developed using phenotypes from the last time-point scored or the AUDPC values, meaning a single time point was adequate to evaluate the crown rust susceptibility of the spaced plants. Much of the predictive ability comes from markers capturing familial relationships, highlighted by the observation that there was no drop in predictive ability when going from the entire marker set down to only 5% (10,878) of the marker set. Accuracy due to genetic relationships will decay rapidly over generations whereas accuracy due to LD will persist. Using a GWAS we attempted to identify and rank markers in LD with QTL. This enabled a small panel of markers to be identified that had higher predictive ability than the same number of randomly selected markers, and had predictive abilities close to those achieved with the entire marker set.

## Additional files

Additional file 1 **Figure S1**. Predictive ability and bias for crown rust using various algorithms for genomic prediction. (PDF 101 kb)

Additional file 2 **Table S1**. List of markers associated with crown rust resistance based on genome wide association studies in complete population and IBERS material. (XLSX 14 kb)

Additional file 3 **Table S2**. List of genomic scaffolds where all the significant markers from genome wide association studies were located. Scaffolds were placed onto linkage group with the aid of Genome Zipper [59]. (XLSX 11 kb)

Additional file 4 **Table S3**. List of predicted proteins on the genomic scaffolds. Markers located on these scaffolds were associated with crown rust resistance. BLAST was done on the predicted protein sequences using PLAZA [62] to obtain the gene function. (XLSX 17 kb)

Additional file 5 **Figure S2**. Among-and-within-full-sib-family selection that incorporates an inexpensive genotyping assay to implement within-family selection using a high selection intensity. (PDF 227 kb)

## Abbreviations

AUDPC: Area under disease progress curve
GEBV: Genomic estimated breeding value
GS: Genomic selection
GWAS: Genome wide association study
LD: Linkage disequilibrium
QTL: Quantitative trait locus
