# Supplementary figures and images for: Genomic prediction of crown rust resistance in Lolium perenne
Source: BMC Genet. 2018 May 29;19:35. doi: 10.1186/s12863-018-0613-z (PMC5975627; doi:10.1186/s12863-018-0613-z)

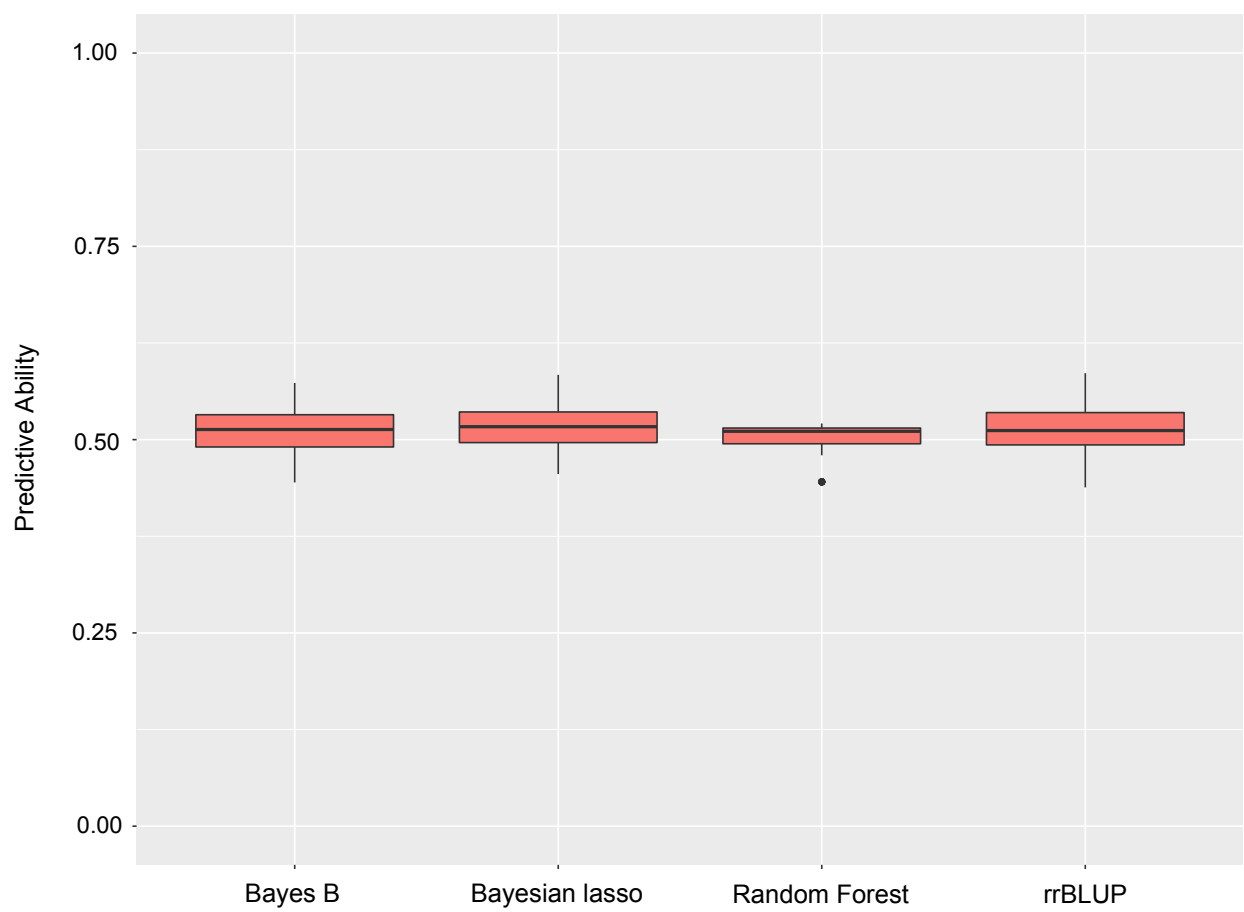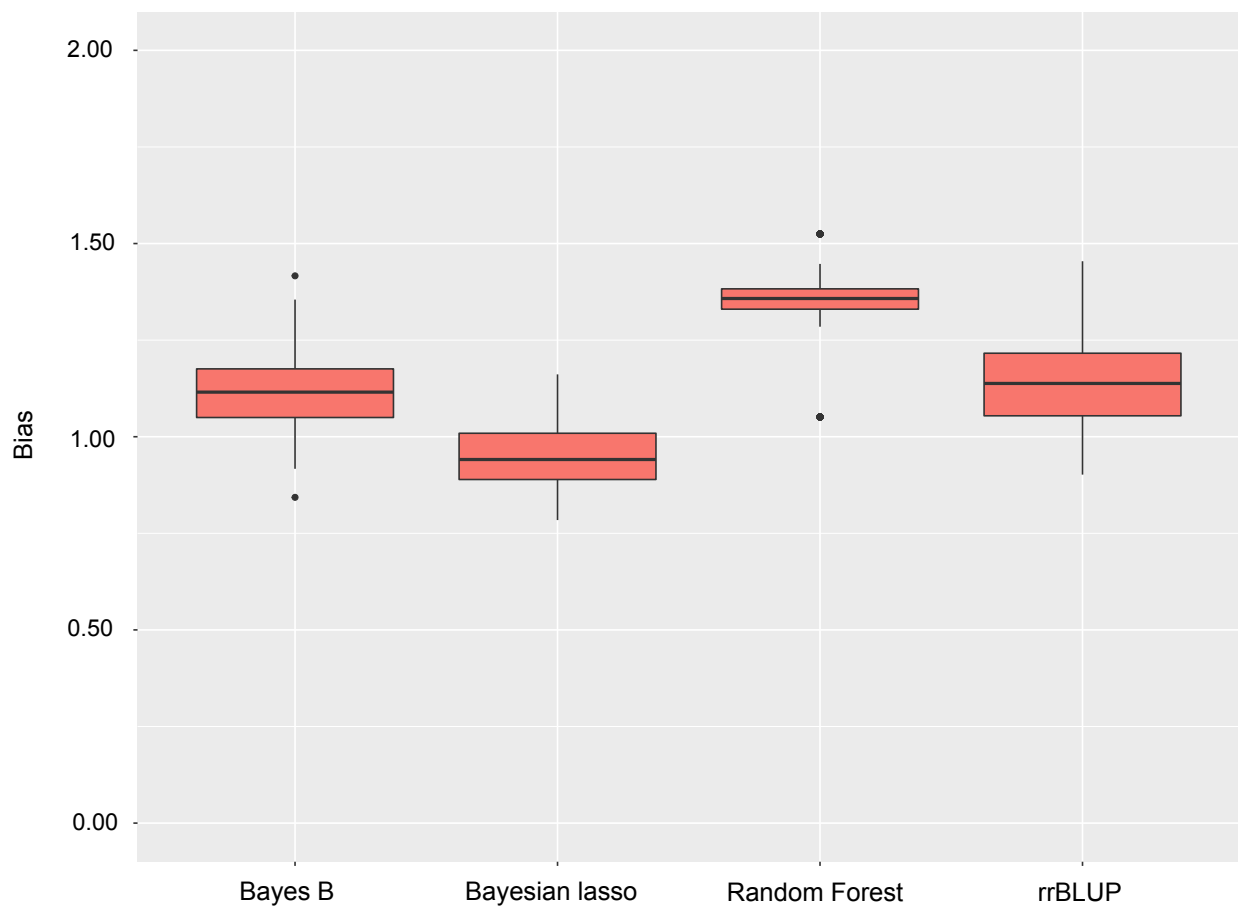

Supplement: Supplementary file 1 — Figure S1. Predictive ability and bias for crown rust using various algorithms for genomic prediction. (PDF 101 kb) [file 12863_2018_613_MOESM1_ESM.pdf]
