# Supplementary material for: Genomic prediction of crown rust resistance in Lolium perenne
Source: BMC Genet. 2018 May 29;19:35. doi: 10.1186/s12863-018-0613-z (PMC5975627; doi:10.1186/s12863-018-0613-z)
Supplement: Supplementary file 5 — Figure S2. Among-and-within-full-sib-family selection that incorporates an inexpensive genotyping assay to implement within-family selection using a high selection intensity. (PDF 227 kb) [file 12863_2018_613_MOESM5_ESM.pdf]

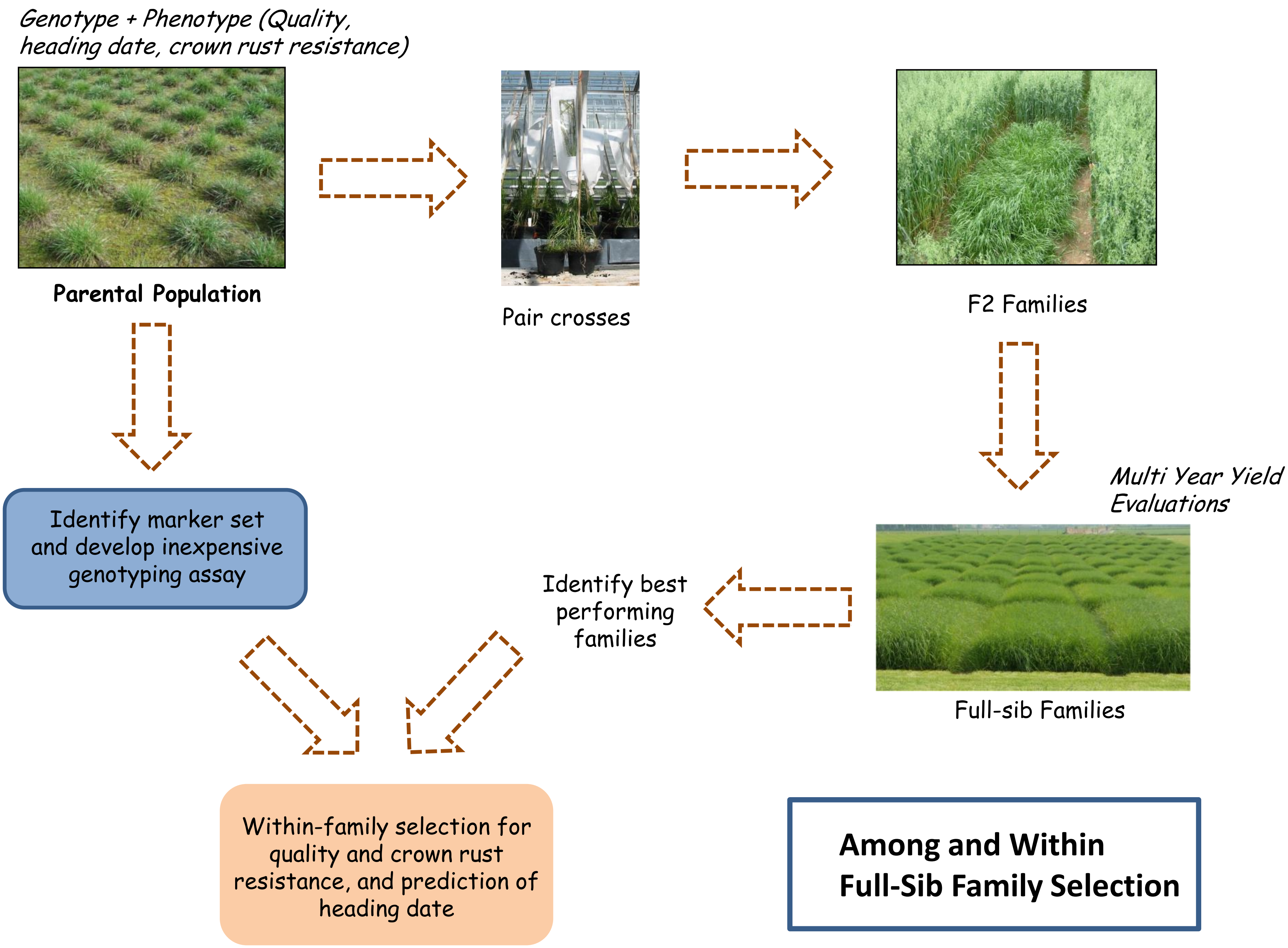
Genotype + Phenotype (Quality, heading date, crown rust resistance)
Parental Population
Pair crosses
F2 Families
Identify marker set and develop inexpensive genotyping assay
Multi Year Yield Evaluations
Identify best performing families
Full-sib Families
Within-family selection for quality and crown rust resistance, and prediction of heading date
Among and Within Full-Sib Family Selection
